# Functionalized Nanoparticles Activated by Photodynamic Therapy as an Antimicrobial Strategy in Endodontics: A Scoping Review

**DOI:** 10.3390/antibiotics10091064

**Published:** 2021-09-02

**Authors:** Pablo Betancourt, Nadia Brocal, Eulàlia Sans-Serramitjana, Carlos Zaror

**Affiliations:** 1 Center for Research in Dental Sciences (CICO), Endodontic Laboratory, Faculty of Dentistry, Universidad de La Frontera, Temuco 4780000, Chile; n.brocal01@ufromail.cl; 2 Department of Integral Adultos, Faculty of Dentistry, Universidad de La Frontera, Temuco 4780000, Chile; 3 Scientific and Technological Bioresource Nucleus (BIOREN), Universidad de La Frontera, Temuco 4780000, Chile; eulalia.sans@ufrontera.cl; 4 Department of Pediatric Dentistry and Orthodontics, Faculty of Dentistry, Universidad de La Frontera, Temuco 4780000, Chile; 5 Center for Research in Epidemiology, Economics and Oral Public Health (CIEESPO), Faculty of Dentistry, Universidad de La Frontera, Temuco 4780000, Chile

**Keywords:** nanoparticle, photosensitizer, antimicrobial photodynamic therapy, root canal infection, endodontics

## Abstract

The eradication of endodontic pathogens continues to be the focus of the search for new root canal system (RCS) disinfection strategies. This scoping review provides a comprehensive synthesis of antimicrobial photodynamic therapy (aPDT) using nanoparticles (NPs) as an alternative to optimize RCS disinfection. A systematic search up to March 2021 was carried out using the MEDLINE, EMBASE, Scopus, Lilacs, Central Cochrane Library, and BBO databases. We included studies focused on evaluating the activation of NPs by aPDT in inoculated root canals of human or animal teeth or bacterial cultures in the laboratory. The selection process and data extraction were carried out by two researchers independently. A qualitative synthesis of the results was performed. A total of seventeen studies were included, of which twelve showed a substantial antibacterial efficacy, two assessed the substantivity of the disinfection effect, and three showed low cytotoxicity. No adverse effects were reported. The use of functionalized NPs with photosensitizer molecules in aPDT has been shown to be effective in reducing the bacteria count, making it a promising alternative in endodontic disinfection. Further studies are needed to assess the development of this therapy in in vivo conditions, with detailed information about the laser parameters used to allow the development of safe and standardized protocols.

## 1. Introduction

The success of endodontic disinfection depends on the eradication of microbes and their by-products from the root canal system (RCS) [1]. Bacterial biofilm is considered to be the main etiological cause of primary and secondary/persistent infection [2]. Existing endodontic treatment strategies have proved insufficient to reduce endodontic pathogenic microorganisms below detection limits [3,4]. The unpredictable nature of the anatomy of the RCS, which consists of accessory canals, isthmuses, lateral canals, apical deltas, and recesses from C-shaped or oval/flattened canals, hinders the adequate action of antiseptic solutions [5,6]. New advanced disinfection approaches are therefore required for the effective eradication of microbial biofilms in endodontic infections. Antimicrobial photodynamic therapy (aPDT) is a promising alternative therapy proposed to combat a broad spectrum of endodontic biofilm-mediated infectious diseases [7,8,9]. It is of particular interest because of its simple approach, a visible light source of a suitable wavelength, generally a low-intensity diode or light-emitting diode (LED), is absorbed by a non-toxic photosensitizer (PS) [10]. The light sources cited for aPDT in the literature are laser, light-emitting diodes (LED) and halogen lamps [11,12]. Of these the most commonly used is the diode laser, due to its portability, ease of handling and low cost compared to other lasers [13]. Furthermore, the range of wavelengths emitted by diode laser coincides with the electronic absorption spectrum of most of the PS available [14]. Phenothiazines (synthetic non-porphyrin compounds), such as methylene blue (MB) and toluidine blue O (TBO), are the most widely studied PS in endodontics [12]. The main disadvantage of conventional PS is their poor solubility in water [15]; an uncontrollable drug-release profile, poor target selectivity, and low extinction coefficient also hamper their clinical action [16]. Therefore, a promising approach to improve the performance of PS within RCS is to encapsulate them in nanostructured materials [17]. In recent works, indocyanine green (ICG) [18,19], rose bengal (RB) [20,21] and erythrosine (ER) [22] have been cited as PS with optimal properties for nanoencapsulation and incorporation into aPDT in endodontic treatment. Nanoparticles (NPs) are defined as submicroscopic particles between 1 nm and 100 nm in size [13,23]. It has been seen that NPs can potentially improve the therapeutic efficacy of pharmaceuticals by promoting better bioavailability, serum stability, and pharmacokinetics of the drug [24]. According to the literature, nano-formulations provide better penetration and allow slow, controlled release of active ingredients at target sites [25]. Their most important feature is their large external surface area, as well as their high surface/volume ratio, factors that determine their physicochemical properties [26]. Different combinations irrigant/sealer can cause subsequent changes in the interfacial shear strength (ISS) of dentin, which may subsequently affect the post-restoration process in the root canal [27]. However, it has been shown that irrigation with NPs does not negatively interfere with the hardness and elastic modulus of dentin [28] or the bond strength and permeability of the interface of bonded fiberglass posts with resin [29]. The use of PS-loaded NPs is a modern strategy in endodontics; it is considered to increase the antimicrobial efficacy of aPDT [30,31]. In recent years, several PSs associated with polymeric [20,21,32,33,34,35,36,37], metallic [18,19,38], and carbon-based NPs [17,39] have been tested in vitro, ex vivo, or in vivo in combination with aPDT. Published studies on the antibacterial action of PS-based NPs complexes and aPDT are remarkably heterogeneous and show great variability in their respective methodologies. This is particularly true due to differences in the PS agents and types of NP used, light activation parameters, bacterial organization (planktonic cells or biofilm) and different bacterial species; this heterogeneity makes it difficult to determine the potential benefit of this new therapeutic combination. Thus, the aim of this scoping review was to address the current status of new PS-loaded NPs complexes used for antimicrobial purposes in endodontics when activated by aPDT.

## 2. Results

### 2.1. Selection of Sources of Evidence

**Figure 1:**
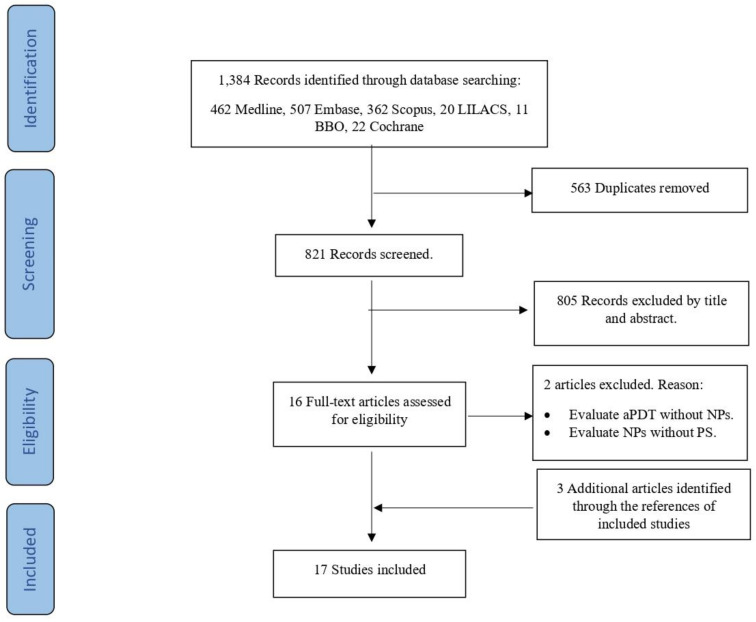
Flow diagram of selection of sources of evidence. aPDT, antimicrobial photodynamic therapy; NPs, nanoparticles; PS, photosensitizer.

A total of 1384 articles were identified from searches of electronic databases, from which 563 duplicates were eliminated. Of the 821 remaining, 805 were excluded based on the title and abstract, and 16 full text articles were retrieved and assessed for eligibility. Of these, two were excluded because they evaluate aPDT without NPs, or evaluate NPs without PS. Three additional articles were included after a manual search of the reference, giving a final total of 17 articles considered eligible for this scoping review. A flow diagram of the selection process is shown in Figure 1.

#### 2.1.1. Characteristics of Sources of Evidence

The main characteristics of the articles included are summarized in Table 1. They were published between 2010 and 2020. Eight studies were from Canada, four from Iran, two from the USA, and one each from Taiwan, India, and Turkey. According to the study design, ten articles were in vitro, five ex vivo, one in vivo, and one incorporated both ex vivo and in vivo designs. The distribution of NPs and PS used were as follows: three articles studied silver NPs (AgNPs), two of which used toluidine blue (TB) as PS, and one used ICG. Chitosan (CS) NPs were analyzed by eight articles, five with rose bengal (RB), three with methylene blue (MB) and RB, and one with erythrosine (ER). Nano-graphene oxide (NGO) NPs were evaluated in two articles: one used ICG, and the other curcumin. The following compounds were each treated in one study: nano-metal organic frameworks (MOFs)-ICG, silica NPs (SiO_2_–NH_2_)-RB, and poly (lactic co-glycolic acid) (PLGA) NPs-MB. Turning to the lasers and their wavelengths, seven articles utilized a diode laser at wavelengths of 50, 620, 665, and 810 nm, three articles a non-coherent light at 540 and 660 nm, two a broad-spectrum lamp at 540 and 660 nm; one a white light source at 540 nm and 660 nm; one a green light source at 540 nm; and one article used a high-power LED array at 540 nm. Two articles did not mention the laser type, although they did mention the wavelengths used, 540 and 630 nm. The duration of light application in a single activation was from 30 s to 60 min, with an average of 9.0 min.

#### 2.1.2. Critical Appraisal within Sources of Evidence

In the overall assessment, 12 articles (70.6%) were considered reliable without restrictions, and five (29.4%) were classified as reliable with restrictions. Table S1 shows the methodological quality for each article. In the “test substance identification” criterion group, two articles (11.8%) did not report the purity of the substance or information on its physicochemical properties; not reporting this information is prejudicial to the transparency of the experiments performed and may affect the quality of their results. In the second criterion group “test system characterization”, all articles (100%) were rated with the highest score. The third criterion group evaluates the “study design description”; five articles (29.4%) did not report the minimum information required in the “description of study design”, and were thus classified as reliable with restrictions. Most of the studies did not describe laser application. Two articles (11.8%) did not mention the type of laser used, although the wavelength used was mentioned. In five articles (29.4%) the time of light application was not mentioned. Thirteen articles (76.5%) did not mention the number of light activations. Twelve articles (70.6%) did not describe how the light was applied. The critical evaluation tool considered only the frequency and duration of exposure as minimum information required. However, all the above-mentioned could lead to a misinterpretation of the study results. It is important to emphasize that the basis of aPDT is light exposure; therefore, a detailed description of this step is imperative. In the fourth criterion group “study results documentation”, all the articles were rated with the highest score. The fifth and final criterion was “plausibility of study design and data”; only one article (5.9%) did not score the maximum for this criterion due to selective outcome reporting.

### 2.2. Synthesis of Results

#### 2.2.1. Antibacterial Efficacy

Twelve studies evaluated the efficacy of aPDT through the colony-forming units (CFU/mL), percentage of reduction in colony count (% RCC), bacterial survival (log CFU mL^−1^), or percentage of biofilm formation. The main results are shown in Table 2. All groups of PS-based NPs achieved an efficacy greater than 90%, except for the MOFs-ICG groups [19], which showed an effectiveness up to 62.67%. Total bacterial eradication was achieved by seven studies, four evaluated CSRB-NPs [20,35,37,41], one evaluated ER-CS [22], one evaluated AgNPs-TBO [42], and the other evaluated SiO_2_–NH_2_–RB [40]. The studies also showed an efficacy greater than 90% compared to laser alone [18], PS plus aPDT (MB and RB) [20,40,41], and NPs alone (AgNPs) [18].

#### 2.2.2. Penetration Capability

One study evaluated the penetration of functionalized NPs into the bacterial biofilm, although this photoactivation was not evaluated [34]. Shrestha et al. [34] evaluated the uptake of CSRB-NPs and RB in the three-dimensional biofilm structure under confocal laser scanning microscopy (CLSM) in an in vitro study design. It was found that 1 mL of CSRB-NPs for 15 min penetrated more deeply (52 μm) into the 7-day-old *E. faecalis* biofilm with a higher uptake than RB under the same conditions.

#### 2.2.3. Substantivity of the Disinfecting Effect

Two studies assessed CSRB-NPs, RB, or MB efficacy in the presence of bovine serum albumin (BSA) or pulp tissue. The efficacy was evaluated immediately after aPDT, and 24 h after aPDT, based on bacterial survival percentage. It was observed that total bacterial eradication was achieved 24 h after CSRB-NPs activation. In contrast, RB and MB showed high bacterial survival 24 h after treatment (Table 3).

#### 2.2.4. Adverse Effects and Possible Toxicity in Adjacent Tissues

None of the included studies reported adverse effects in adjacent tissues. Three authors studied possible toxicity in adjacent tissues. Cell viability was assessed in macrophage cells, human embryonic kidney cell line, and in mouse fibroblast cells treated with NPs functionalized with PS. Low cytotoxicity of aPDT was noted when CSRB-NPs [35,36] and TBO–AgNP were evaluated. CSRB-NPs also exhibited no cytotoxicity prior to light irradiation; however, they presented a certain level of cytotoxicity in one study after aPDT. No such increase in the level of cytotoxicity was observed with RB [35] (Table 4).

#### 2.2.5. Antibacterial Efficacy of Functionalized NPs in the Absence of Light

Light activation is the principle of aPDT; however, in some studies a disinfecting effect was noted even before light application. This ability to become activated in the absence of light is known as the dark toxicity of substrates [22]. Two studies using CSRB-NPs [35] and ER/CS-NPs [22] showed decreased bacterial survival in the dark. In the case of CSRB-NPs, the effect increased with increasing concentration of the agent. One study evaluated Al-101, Fe-88, and Fe-101 functionalized with ICG. A reduction in CFUs was achieved by all three groups [19] (Table 5).

#### 2.2.6. Impact of Tissue Inhibitors on Antibacterial Efficacy

Dentin, dentin matrix, and lipopolysaccharides (LPS) have a smaller inhibitory effect than pulp tissues and BSA [20,35]. There was an important inhibitory effect of pulp, which supports the assertion that its elimination should be a priority. However, even in the presence of pulp tissue, complete efficacy was achieved by CSRB-NPs after 24 h. By contrast, RB or MB were unable to eradicate bacteria completely in the presence of pulp tissue. When BSA was evaluated, complete bacterial eradication was observed using CSRB-NPs. By contrast, only 3% bacterial eradication was achieved when MB was used [20] (Table 3).

#### 2.2.7. Ability of PS-Based NPs to Neutralize Pro-Inflammatory Agents

The ability of antimicrobial agents to neutralize LPS was explored in two studies by Shrestha et al. [21,36] in an in vitro and in vivo study design, respectively. The in vitro study evaluated the neutralization of LPS by CSRB-NPs and MB. The production of nitric oxide (NO), interleukin-6 (IL-6), and tumor necrosis factor-alfa (TNF-α) by macrophages was used to determine the inflammatory potential of LPS. The results showed that aPDT using CSRB-NPs and MB was effective in neutralizing LPS, subsequently reducing their inflammatory potential [36]. In the in vivo study, neotissue formation was assessed on LPS-contaminated teeth implanted into guinea pigs’ mandibles. The best result was achieved by the combination therapy based on NaOCl and photoactivated CSRB-NPs. Treatment with NaOCl alone did not achieve total elimination of LPS.

## 3. Discussion

The aim of our scoping review was to address the current status of new PS-NP complexes used for antimicrobial purposes in endodontics when activated by aPDT. After the selection process, 17 studies met our inclusion criteria. These studies showed substantial effectiveness in antibacterial efficacy, penetration ability and adequate substantivity. No adverse effects were reported.

### 3.1. Antibacterial Efficacy

The high antibacterial efficiency shown by PS-NPs may be due to the physico-chemical characteristics of the NPs, including their ultra-small size, higher chemical reactivity, and large surface area/mass ratio [35,43]. The different nanocomplexes studied have optimal adhesion capacities to the biofilm and permeability of the bacterial cell membrane, which translates into improved effectiveness of aPDT [22,44]. They also exhibited antibacterial properties, such as a high affinity for the bacterial cell membrane and ability to penetrate deeply into biofilms, thus provoking effective disruption of their structure. All of these characteristics are considered relevant in a clinical setting [20,34]. Furthermore, a transmission electron microscopy (TEM) study determined that the localization of PS-loaded NPs was mainly visualized in the bacterial cell wall, corroborating previous reports [22,34]. It should be noted that the concentration of PS when incorporated into NP is one-fifth of its concentration in conventional aPDT, reducing the probability of cytotoxicity and tooth discoloration [39]. The studies included in this scoping review confirm that the nanostructure makes PS more stable, and improves its antimicrobial properties.

### 3.2. Penetration Capability

The penetration capability into bacterial biofilms is a significant problem when total microbial eradication is sought. The conjugation of PS with nanoscale cationic particles such as CS also influenced physicochemical interaction with the cell wall, and contributed to the anti-biofilm effect [37]. CS has shown the ability to intercalate with extracellular DNA and irreversibly modify the biofilm structure [45]. In this regard, it is recommended that biofilms of three weeks’ maturation be used in the study methodology to test new antimicrobial therapies, since they have already reached abundant EPS thickness [46]. Although the antimicrobial results were favorable, further studies are needed to determine the penetration of other NP-PS combinations.

### 3.3. Substantivity of the Disinfecting Effect

A prolonged antimicrobial effect is a desirable characteristic when evaluating a disinfectant agent in endodontics. The articles included showed a considerable reduction in the bacterial survival percentage 24 h after PS-NP light activation, even in the presence of tissue inhibitors [20,41]; however, this prolonged efficacy was not present in the PS groups alone [20,35]. The results suggested that treating the dentin surface with NPs could prevent bacterial adherence, thus avoiding bacterial recolonization and the formation of biofilms [34]. However, it must be considered that the light delivery system into the RCS through a thin optical fiber must be effective, allowing the light to be transmitted homogeneously even in the most apical third of the root [47].

### 3.4. Adverse Effects and Possible Toxicity in Adjacent Tissues

From a clinical point of view, the antimicrobial action of photoactivation of a PS should exhibit extensive destruction of pathogens with minimal damage to host tissues [22]. None of the articles included mentioned any adverse effects; however, dental staining and discoloration have been reported as an adverse effect of conventional aPDT when MB is used as PS [48,49], being more marked in an application interval of 10 min than one of 5 min [50]. The use of a nanocarrier to transport the PS clearly reduces tooth discoloration, favoring the clinical application of aPDT in endodontics. The toxicity in adjacent tissues was evaluated in three different cell lines, reaching low [35,38] or even zero toxicity [36]. Moreover, the concentration of TBO which reduces 99.9% of bacterial cells does not show any significant cytotoxic effect on human fibroblasts in culture [51].

### 3.5. Antibacterial Efficacy of Functionalized NPs in the Absence of Light

Chen et al. [22] indicated that an ideal aPDT should induce the antimicrobial effect only upon light illumination. This is proposed as a key factor for allowing penetration into dentinal tubules and correct distribution inside the bacterial biofilm. It has been observed that PS does not exhibit toxicity in the dark [52] and its action is dependent on the application of light irradiation [31,38]. This could potentially cause beneficial effects if we consider the limitations on access of light to all the anatomical complexities of root canals. Controlled activation in the absence of light may enhance disinfection in those hard-to-reach areas. Although activation in the absence of light is the subject of discussion among the authors, the studies included in this review showed that the functionalized NPs used present certain bacterial killing rates in the absence of light (Table 5) [19,22,35]. More studies are needed to develop this topic.

### 3.6. Impact of Tissue Inhibitors on Antibacterial Efficacy

Different organic tissues present in root canals, such as necrotic pulp tissue, dentin debris, bacterial endotoxins, bacterial LPS, and serum, appear to play an important role in the effect of aPDT [53]. These are different tissue inhibitors that impact negatively on the interaction of PS and the bacterial cell and reduce the half-life of the singlet oxygen produced in aPDT [54]. Thus, they significantly decrease the antibacterial effectiveness of aPDT [37]. Two studies have shown that the action of photoactivated cationic MB and anionic RB dyes is limited in the presence of BSA and pulp tissue, showing little or no effectiveness at 24 h [20,35]. However, it has been seen that incorporating a photosensitizer into nanostructures increases their antimicrobial effectiveness when activated by light with a certain wavelength in the presence of tissue inhibitors. By contrast, photoactivated MB and RB were not able to remove bacterial cells successfully [20]. The study models employed to evaluate endodontic disinfection are commonly far removed from natural conditions. This explains the use of monospecific biofilms, which grow on a sterilized tooth or well plates without the interaction of any organic material [17,18,39]. The incorporation of tissue inhibitors or bacterial toxins into the study model could improve the representation of clinical conditions, as a realistic way of analyzing antimicrobial substances and demonstrating results close to reality in the early stages of an investigation [20,41]. 

### 3.7. Ability of PS-Based NPs to Neutralize Pro-Inflammatory Agents

Effective disinfection of the RCS is achieved with the removal of both bacterial biofilms and their by-products, which provides the basis for regeneration and healing of the affected apical tissue [3]. However, conventional chemo-mechanical therapy is ineffective in many cases in eliminating/inactivating bacterial modulins such as endotoxins/LPS from infected root dentin [55]. The activation of CS-NPs has been shown to be effective in neutralizing LPS [56]. Furthermore, the aPDT activation of CSRB-NPs has been shown to be effective in the inactivation of bacterial endotoxins, particularly of LPS obtained from *P. aeruginosa* [36]. Therefore, due to their antibacterial effectiveness, plus their ability to inactivate endotoxins, CSRB-NPs activated by aPDT could be an effective alternative for reducing the expression of inflammatory cytokines from macrophage cells [36]. Furthermore, in an in vivo experimental design, it was observed that photoactivated functionalized NPs (CSRB-NPs) favored the formation of healthy tissue, suggesting the effective inactivation of LPS bound to dentin, corroborating results reported previously [21].

### 3.8. Limitations

The present scoping review has some limitations. First, although we were systematic in our review, it is possible that we may have failed to identify all studies. However, we believe that this has been minimized by the sensitive search strategy used, the additional search of references by hand, and the double independent review process followed. In addition, the grey literature was systematically reviewed. Second, we only selected studies published in English, Spanish, or Portuguese; therefore, our results could have missed important studies written in other languages. Third, quality assessment was hampered by poor reporting in the studies included. We tried to contact the authors for more information but did not get a satisfactory response. One of the main areas that is not clear due to the lack of information is that the protocols used in light activation; how the energy activates the PS, and for how long could affect the effectiveness of the therapy. It is not feasible for a clinical therapy to use long activation times. Consequently, results of studies with excessively high light application times could lead to lack of clinical applicability, and misrepresentation of the real effectiveness a therapy could achieve.

### 3.9. Implications for Practice and Research

The use of PS-loaded nanostructures will clearly help to reduce the worldwide antibiotic resistance generated in recent decades. Their easy application in the endodontic field would imply an improvement in the success rate of treatments, improving patients’ quality of life. In order to achieve a real therapeutic impact, the market price of nanocomplexes needs to be affordable for most clinicians. However, further studies are needed to assess the development of this therapy in in vivo conditions. Future research must include detailed information about the laser parameters employed to provide evidence that allows the development of safe protocols.

## 4. Materials and Methods

### 4.1. Protocol and Registration

This scoping review was reported according to the Preferred Reporting Items for Systematic Reviews and Meta-Analyses Extension for Scoping Reviews [57]. The protocol is available and can be accessed at https://osf.io/ztv9u/?view_only=759adc2a837346f3b31c031cb1704280 (accessed on 29 July 2021).

### 4.2. Eligibility Criteria

We included primary studies focused on evaluating the activation of NPs by aPDT in inoculated root canals of human or animal teeth (ex vivo or in vivo) or bacterial cultures in laboratory (in vitro) against endodontopathogenic microorganisms, published in English, Spanish, or Portuguese. There were no restrictions on publication dates. Secondary studies, non-experimental studies, editorials, letters, case reports, case series, dissertations, expert opinions, and book chapters were excluded.

### 4.3. Sources of Information and Search Strategy

A systematic search of the literature up to 22 March 2021 was conducted using the Medline, Embase, Scopus, Lilacs, Central Cochrane Library, and BBO databases. The search strategy used in Medline was: ((nanoparticle* OR NPs OR “Nanoparticles”[Mesh])) AND ((root canal OR endodontic OR “Root Canal Therapy”[Mesh] OR pulpectomy OR “Pulpectomy”[Mesh])). The adapted search strategies performed in the other databases are shown in the Supplementary Materials (Table S2). The gray literature was explored on 22 March 2021 consulting the OpenGrey information system (http://www.opengrey.eu/ accessed on 29 July 2021) and EThOS British Library (https://ethos.bl.uk/ accessed on 29 July 2021) using the following terms: root canal, endodontic, nanoparticles, photodynamic therapy. Additionally, (https://clinicaltrials.gov/ accessed on 29 July 2021) was consulted up to March 2021 to identify registered clinical trials. The reference lists of selected articles were screened to detect other potentially eligible studies.

### 4.4. Selection of Sources of Evidence

All references identified were exported into the Research Information Systems (RIS) file and uploaded into the EndNote software, where duplicates were automatically eliminated. Articles were then exported into the Rayyan online software (https://rayyan.qcri.org/ accessed on 29 July 2021) for selection on 22 March 2021. The blind mode was activated so that each reviewer’s activity would be hidden from the others. Two reviewers (N.B. and P.B.) independently performed the selection of the studies by title and abstract, and then by full text according to the eligibility criteria. If there was a discrepancy, a consensus was reached. The reviewers were not blinded to the authors or journals. The reasons for exclusions were recorded.

### 4.5. Data Charting Process

Data extraction was performed by two reviewers (N.B. and P.B.). One reviewer collected the required information from the selected articles and the second crosschecked all the data. The following items were extracted from each article using a predefined excel form: study identification information, type of study, study objective, number of samples, study model, description of the study model, microorganism employed, organizational form of the bacteria studied, type of light used (laser name and wavelength), laser parameters, NP and PS used, effectiveness of treatment, control and experimental group (number of samples and components used), duration of light application and number of activations, diameter of the tip, tip work position, and principal conclusions of the study. Disagreements were resolved through discussion and mutual agreement between the two reviewers.

### 4.6. Critical Appraisal

Selected articles were analyzed by two reviewers (N.B. and P.B.) independently according to the Toxicological data Reliability Assessment Tool (ToxRTool) [58] using a predetermined Microsoft Excel^®^ file. This tool consists of two different sections, one for in vivo and one for in vitro data. The ex vivo analysis was included in the in vitro section. The tool includes 21 criteria for in vivo studies and 18 criteria for in vitro studies. In addition, criteria are subdivided into five groups: I. test substance identification; II. test system characterization; III. study design description; IV. study results documentation; and V. plausibility of study design and data. Each criterion can be assigned either a “1” (one point), i.e., “criterion met”, or a “0” (no point), i.e., “criterion not met”. The total points assigned to a given study led to the proposal of a reliability category (1 to 3). For in vivo studies, a sum of 18–21 points put the study in the first reliability category (“reliable without restrictions”), 13–17 points in the second category (“reliable with restrictions”), and less than 13 points in the third category (“not reliable”). In vitro studies awarded 15–18 points were placed in the first category, 11–14 points in the second category, and less than 11 in the third category. For both types of studies, there was a fourth category (“not assignable”) if the documentation was insufficient (reviews, handbooks, other secondary sources). In addition, there were minimum information requirements. If these criteria were not met, the tool assigned a lower data reliability category, regardless of the total score obtained [58].

### 4.7. Synthesis of Results

Finally, the results were synthesized following the recommendations of Green et al. [59]. A narrative overview model, which is a broad narrative synthesis of formerly published studies, was constructed. Tables were used to present information on the antibacterial efficacy of the therapy, substantivity of the disinfecting effect and impact of inhibitors, toxicity in adjacent tissues, and antibacterial efficacy in the absence of light. When articles showed more than one efficacy result, the mean and standard deviation of the numerical data were calculated. If the article only showed its results information in graphs, images, or figures, the numerical data were extracted using the Web Plot Digitizer tool 4.2 for Mac software.

## 5. Conclusions

The use of functionalized NPs with PS molecules in aPDT has been shown to be effective in reducing the bacteria count, making it a promising alternative in endodontic disinfection. Further studies are needed to assess the development of this therapy in in vivo conditions, with detailed information about the laser parameters used to allow the development of safe and standardized protocols.

## Figures and Tables

**Table 1:** Summary of studies included.

Reference	Study Type	Microorganisms	NPs	PS	Bacterial Organization	Bacterial Incubation Time
Guo et al. 2010 [40]	In vitro	*S. aureus* and *S. epidermidis*	SiO_2_–NH_2_–RB	RB	Planktonic	20 h
Pagonis et al. 2010 [32]	In vitro, ex vivo	*E. faecalis*	PLGA	MB	Biofilm	3 days
Chen et al. 2012 [22]	In vitro	*S. mutans*, *Pseudomonas aeruginosa* and *Candida albicans.*	CSNPs	ER	Biofilm/ planktonic	24 h–48 h/ overnight
Shrestha et al. 2012 [37]	In vitro	*E. faecalis* and *P. aeruginosa*	CSRB-NPs	RB, MB	Biofilm/planktonic	7 days/not mentioned
Shrestha et al. 2012 [41]	In vitro	*E. faecalis*	CSRB-NPs	RB	Biofilm/planktonic	7 days
DaSilva et al. 2013 [33]	Ex vivo	*E. faecalis.*	CSNPs	RB	Biofilm	7 days
Shrestha et al. 2014 [20]	In vitro	*E. faecalis,* and LPS from *Escherichia coli*	CSRB-NPs	RB, MB	Planktonic	Overnight
Shrestha et al. 2014 [34]	In vitro	*Streptococcus oralis*, *Prevotella intermedia,* and *Actinomyces naeslundii*	CSRB-NPs	RB	Biofilm	21 days
Shrestha et al. 2014 [35]	In vitro	*E. faecalis*	CSRB-NPs	RB	Biofilm	21 days
Shrestha et al. 2015 [36]	In vitro	LPSs from *P. aeruginosa*	CSRB-NPs	MB	Not applicable	Not applicable
Afkhami et al. 2016 [18]	Ex vivo	*E. faecalis*	AgNPs	ICG	Biofilm	4 weeks
Misba et al. 2016 [38]	In vitro	*S. mutans*	AgNPs	TBO	Biofilm/ planktonic	Not mentioned
Akbari et al. 2017 [39]	In vitro	*E. faecalis*	NGO	ICG	Biofilm/ planktonic	24 h/4–5 h
Golmohamadpour et al. 2018 [19]	Ex vivo	*E. faecalis*	MOFs (Fe-101, Al-101 and Fe-88)	ICG	Biofilm/planktonic	2 weeks/24 h
Shrestha et al. 2018 [21]	In vivo	LPSs from *P. aeruginosa*	CSRB-NPs	RB	Not applicable	Not applicable
Aydin et al. 2020 [42]	Ex vivo	*E. faecalis*	AgNPs	TBO	Biofilm	21 days
Ghorbanzadeh et al. 2020 [17]	Ex vivo	*E. faecalis*	NGO	Cur	Biofilm	4 weeks

AgNPs, silver NPs; ICG, indocyanine green; NGO, nano-graphene oxide NPs; CS, chitosan NPs; ER, erythrosine; RB, rose Bengal; MOFs, metal organic frameworks; SiO_2_–NH_2_, Silica; TBO, toluidine blue-o; PLGA/MB, poly (lacticco-glycolic acid) NPs; MB, methylene blue; Cur, curcumin; LPS, lipopolysaccharides.

**Table 2:** Treatment efficacy data.

Reference	NPs/PS	RT; RD	Efficacy
Guo et al. 2010 [40]	SiO_2_–NH_2_/RB	40 min; ∼33 J/cm^−2^	VCC: Log 8 CFU/mL reduction
Pagonis et al. 2010 [32]	PLGA/MB	5 min; 30 J/cm^2^	BS (%): 3.3 (Planktonic); 15.2 (Biofilm)
Chen et al. 2012 [22]	CSNPs/ER	Not mentioned; 50 J/cm^2^	VCC: *S. mutans*: Log 7 CFU/mL reduction; *P. aeruginosa*: Log 3.5 CFU/mL reduction; *Candida albicans*: total eradication
Shrestha et al. 2012 [37]	CSNPs/RB	Not mentioned; 20–60 J/cm^2^	BS (%): *E. faecalis*: 2.6 ± 2 * *P. aeruginosa:* 0.8 ± 1.8 *
Shrestha et al. 2012 [41]	CSNPs/RB	X min; 5–60 J/cm^2^	BS (%): no bacterial survival (planktonic)/range from 1.7 to 2.9 (biofilm)
Shrestha et al. 2014 [20]	CSNPs/RB	1.6–3.3 min; 5–10 J/cm^2^	No bacterial survival
Shrestha et al. 2014 [35]	CSNPs/RB	15 min; 2–60 J/cm^2^	BS (%): 4.4 ± 2.8 * (0.1 mg/mL); 2.7 ± 2.4 * (0.3 mg/mL)
Afkhami et al. 2016 [18]	AgNPs/ICG	30 s; 200 mW	RCC (%): 99.12
Misba et al. 2016 [38]	AgNPs/TBO	70 s; 9.1 Jcm^−2^	RBF (%): 69 ± 22.2 *
Akbari et al. 2017 [39]	NGO/ICG	60 s; 31.2 J/cm^2^	RCC (%): 90.6 RBF (%): 99.4
Golmohamadpour et al. 2018 [19]	Fe88/ICG, Al101/ICG, Fe101/ICG	Not mentioned; 31.2 J/cm^2^	BS (%): 45.1, 60.7, 62.7 RBF (%): 37.5, 53.6, 47
Aydin et al. 2020 [42]	AgNPs/TBO	30–60 s; not mentioned	RCC (%): range from 98.8 to 100

RT, radiation time; RD, radiation dose; % RCC, percentage of reduction in colony count; % RBF, reduction biofilm formation; VCC, viable cell counts; % BS, bacterial survival; * M ± SD (mean ± standard deviation).

**Table 3:** Antibacterial substantivity and impact of inhibitors.

Reference	Bacteria	LS	Photosensitization Time	PS-NPs	Inhibitor	LS Energy (J/cm^2^)	Efficacy after aPDT (% BS)	Efficacy 24 h after aPDT (% BS)
Shrestha et al. [20]	Planktonic *E. faecalis*	Broad-spectrum lamp 540 or 660 nm	15 min	CSRB-NPs	Pulp	5	95	26
						10	87	0
					BSA	5	87	16
						10	84	0
				MB	Pulp	5	78	66
						10	79	62
					BSA	5	84	55
						10	77	40
				RB	Pulp	5	95	75
						10	93	47
					BSA	5	85	97
						10	89	96
Shrestha et al. [35]	Planktonic *E. faecalis*	Broad-spectrum lamp 540 ± 15 nm	15 min	CSRB-NPs	BSA	5	75	14
						10	76	0
				RB	BSA	5	91	75
						10	90	80

LS, laser; % BS, bacterial survival; BSA, bovine serum albumin.

**Table 4:** Toxicity in adjacent tissues.

Author	Cell Line	NPs or PS	Time	Cell Viability
Shrestha et al. [35]	Mouse fibroblast	CSRB-NPs	15 min	72.86% cell survival
		RB	15 min	51.23% cell survival
Shrestha et al. [36]	Macrophage	CSRB-NPs	12 h	did not exhibit any toxicity
		MB	12 h	reduction of cell survival not statistically significant
Misba et al. [38]	Human embryonic kidney (HEK-293)	TBO	Not mentioned	>80% cell viability.
		AgNPs	Not mentioned	did not exhibit any toxicity
		TBO–AgNP	Not mentioned	>75% cell viability

**Table 5:** Antibacterial efficacy in absence of light.

Reference	Bacteria	NPs or PS	Time	Results
Chen et al. [22]	Planktonic *S. mutans*	ER⁄CS	12 h	not significant
		CS	12 h	0.5-log ↓
	Planktonic *C. albicans*	ER⁄CS	24 h	not significant
		CS	24 h	1.5-log ↓
Shrestha et al. [35]	Planktonic *E. faecalis*	CSRB-NPs 0.1 mg/ml	15 min	4.5-log ↓
		CSRB-NPs 0.3 mg/ml	15 min	6.5-log ↓
		RB	15 min	not significant
Golmohamadpour et al. [19]	*E. faecalis*	Al-101/ICG	5 min	28.26% ↓
		Fe-88/ICG	5 min	28.55% ↓
		Fe-101/ICG	5 min	41.52% ↓

CS, chitosan NPs; ER, erythrosine; RB, rose bengal; ICG, indocyanine green; Al, aluminum metal organic frameworks; Fe, iron metal organic frameworks.

## Supplementary Materials

The following are available online at https://www.mdpi.com/article/10.3390/antibiotics10091064/s1, Table S1: Critical appraisal details; Table S2: Search strategies performed in electronic databases.
