# Supplementary material for: Functionalized Nanoparticles Activated by Photodynamic Therapy as an Antimicrobial Strategy in Endodontics: A Scoping Review
Source: Antibiotics (Basel). 2021 Sep 2;10(9):1064. doi: 10.3390/antibiotics10091064 (PMC8472326; doi:10.3390/antibiotics10091064)
Supplement: Supplementary file 1 [file antibiotics-10-01064-s001.zip › antibiotics-1340747-supplementary.pdf]

Supplementary material

Table S1. Critical appraisal details.

Reference	Type of Study	I: Test substance identification	II: Test system characterization	III: Study design description	IV: Study results documentation	V: Plausibility of study design and data	Total points	It is considered reliable (yes/no)	Reliability category
Afkhami, 2016[19]	*Ex vivo*	2	3	6	3	2	16	Yes	1
Akbari, 2017[20]	*In vitro*	4	3	5	3	2	17	Yes	1
Chen, 2012[21]	*In vitro*	4	3	4	3	1	15	No	2
DaSilva, 2013[22]	*Ex vivo*	3	3	6	3	2	17	Yes	1
Golmohamadpour, 2018[23]	*Ex vivo*	4	3	4	3	2	16	No	2
Guo, 2010[24]	*In vitro*	4	3	6	3	2	18	Yes	1
Misba, 2016[25]	*In vitro*	4	3	6	3	2	18	Yes	1
Pagonis, 2010[26]	*In vitro-ex vivo*	4	3	6	3	2	18	Yes	1
Shrestha, 2012[27]	*In vitro*	4	3	6	3	2	18	Yes	1
Shrestha, 2014[3]	*In vitro*	4	3	6	3	2	18	Yes	1
Shrestha, 2014[28]	*In vitro*	4	3	5	3	2	17	No	2
Shrestha, 2014[29]	*In vitro*	4	3	5	3	2	17	Yes	1
Shrestha, 2015[30]	*In vitro*	4	3	4	3	2	16	No	2
Shrestha, 2018[31]	*In vivo*	4	5	7	3	2	21	Yes	1
Shrestha, 2012[32]	*In vitro*	4	3	5	3	2	17	No	2
Aydin, 2020[33]	*Ex vivo*	4	3	6	3	2	18	Yes	1
Ghorbanzadeh, 2020[34]	*Ex vivo*	4	3	6	3	2	18	Yes	1

Table S2. Search Strategies performed in electronic databases.

Data base	Search Strategies
Medline	((nanoparticle* OR NPs OR "Nanoparticles"[Mesh])) AND ((root canal OR endodontic OR "Root Canal Therapy"[Mesh] OR pulpectomy OR "Pulpectomy"[Mesh]))
Embase	(nanoparticle* OR nps OR nanoparticle) AND ('root canal' OR endodontic* OR 'endodontic procedure' OR pulpectomy)
Scopus	(TITLE-ABS-KEY (nanoparticle* OR nps) AND TITLE-ABS-KEY ("root canal" OR endodontic* OR "root canal therapy" OR pulpectomy))
LILACS	(tw:(nanoparticle$ OR NPs)) AND (tw:("root canal" OR endodontic$ OR "root canal therapy" OR pulpectomy))
BBO	(tw:(nanoparticula$ OR NPs)) AND (tw:("canal radicular" OR "cavidade pulpar" OR endodontia OR "tratamento do canal radicular" OR pulpectomia))
Central Cochrane Library	#1:MeSH descriptor: [Nanoparticles] explode all trees<br>#2: nanoparticle* OR NPs<br>#3: #1 OR #2<br>#4: MeSH descriptor: [Root Canal Therapy] explode all trees<br>#5: MeSH descriptor: [Pulpectomy] explode all trees<br>#6: "root canal" OR endodontic* OR pulpectomy<br>#7: #4 OR #5 OR #6<br>#8: #3 AND #7
